# Materials Imaging and Dynamics (MID) instrument at the European X-ray Free-Electron Laser Facility

**DOI:** 10.1107/S1600577521001302

**Published:** 2021-02-15

**Authors:** A. Madsen, J. Hallmann, G. Ansaldi, T. Roth, W. Lu, C. Kim, U. Boesenberg, A. Zozulya, J. Möller, R. Shayduk, M. Scholz, A. Bartmann, A. Schmidt, I. Lobato, K. Sukharnikov, M. Reiser, K. Kazarian, I. Petrov

**Affiliations:** a European X-ray Free-Electron Laser Facility, Holzkoppel 4, 22869 Schenefeld, Germany

**Keywords:** XFEL, coherent X-ray scattering, X-ray imaging, dynamics

## Abstract

The Materials Imaging and Dynamics (MID) instrument at the European X-ray Free-Electron Laser (EuXFEL) facility is described. EuXFEL is the first hard X-ray free-electron laser operating in the MHz repetition range which provides novel science opportunities. The aim of MID is to enable studies of nano-structured materials, liquids, and soft- and hard-condensed matter using the bright X-ray beams generated by EuXFEL. Particular emphasis is on studies of structure and dynamics in materials by coherent scattering and imaging using hard X-rays. Commission of MID started at the end of 2018 and first experiments were performed in 2019.

## Abstract 2

The Materials Imaging and Dynamics (MID) instrument at the European X-ray Free-Electron Laser facility is described. Recent commissioning results are discussed to illustrate the potential experimental applications of MID.

## 1. Introduction   

**Figure 1:**
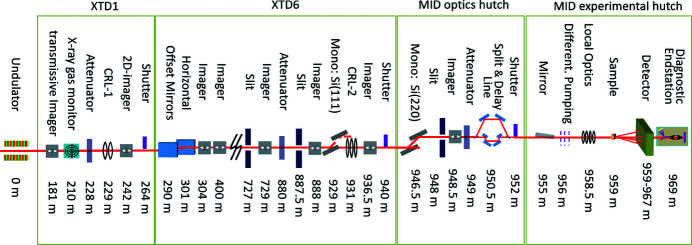
Selection of important components for the MID instrument in the tunnels XTD1/XTD6 and the optics/experimental hutches (OH/EH). The spontaneous radiation apertures SRA (198 m), HIREX spectrometer (360 m), and distribution mirror M3 (390 m) are not shown.

The Materials Imaging and Dynamics (MID) instrument (Madsen, 2011 ▸; Madsen *et al.*, 2013 ▸) belongs to the first generation of instruments built at the European X-ray Free-Electron Laser (EuXFEL) facility in Hamburg, Germany (Altarelli, 2015 ▸; Tschentscher *et al.*, 2017 ▸; Madsen & Sinn, 2017 ▸; Weise & Decking, 2017 ▸; Decking *et al.*, 2020 ▸). EuXFEL is the first hard X-ray laser based on superconducting linac technology operating at MHz repetition rate. Distributing the accelerated electrons over a fan of beamlines allows parallel operation of three undulators (later option of increasing to five). Together with the High Energy Density (HED) instrument, MID receives X-rays from the SASE-2 undulator which is located in the southernmost tunnel of EuXFEL. Current operation constraints are ∼5–25 keV photon energy, ∼10^12^ photons pulse^−1^, and up to 3000 pulses s^−1^ delivered in 10 trains s^−1^ with intra-train repetition rates up to 4.5 MHz. A later increase in the available pulses s^−1^ is expected pending radiation safety verification. The beam is shared between MID and HED, typically switching between instruments every 12 h. This is accomplished by inserting a mirror (M3) which deflects the beam at glancing angles horizontally towards HED. Without M3 inserted the beam continues straight into the MID beamline. The angular difference between the two beamlines is ∼0.15° which yields 1.4 m horizontal separation at the end of the tunnel (∼550 m from M3). This allows installing separate X-ray optics and diagnostics components towards the end of the tunnel without interference between the two beamlines. After exit from the tunnel the beam continues into the experimental hall. MID occupies two adjacent safety enclosures (hutches): the optics hutch (OH) and the experiment hutch (EH) where the beam conditioning and experiments take place, respectively. All instrument devices in the tunnel and the two hutches can be controlled and read out from MID’s control room from which the experiments are conducted. In addition, there are two optical laser hutches (pump–probe laser hutch and instrument laser hutch) and a sample preparation laboratory of relevance for the experiments. These resources are shared between MID and HED. The optical laser setup is currently under installation and commissioning and shall be the subject of a later publication. In the following, the MID instrumentation located in the tunnel, the OH, and EH are described in detail, see Fig. 1 ▸.

## 2. Tunnel components   

The SASE-2 undulator consists of 35 insertion devices in series each of 5 m length that generate the extremely intense pulsed X-ray beam via self-amplified spontaneous emission (SASE) (Abeghyan *et al.*, 2019 ▸). Per definition the zero-point (source point) for distances along the photon beamline is set to the middle of the 33rd insertion device, *i.e.* *z* = 0. In the following all distances refer to this zero point. An important component for MID is the compound refractive lens unit one (CRL-1). It has been described in previous publications (Madsen *et al.*, 2013 ▸; Roth *et al.*, 2014 ▸; Zozulya *et al.*, 2019 ▸) and we repeat the essential features in the following.

### 2.1. CRL-1 unit   

The main purpose of CRL-1 (JJ X-Ray) located at 229 m is collimation of the X-ray beam exiting the SASE-2 undulator. SASE radiation has a very small natural divergence, about 0.5–2 µrad depending on the photon energy and linac parameters (Schneidmiller & Yurkov, 2011 ▸; Sinn *et al.*, 2012 ▸), but with drift sections on the order of 1 km length it would still result in beams with cross sections of 1 mm, or more, at the experimental stations. This is unwanted for many experiments and therefore the CRL-1 unit is employed as beam collimator. It features X-ray lenses mounted on ten actuator arms that can be inserted in any combination into the beam. Concave lenses are focusing X-rays since the refractive index *n* is slightly smaller than unity with 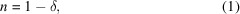


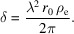
Here λ is the X-ray wavelength, *r*_0_ is the Thomson radius (2.82 × 10^−5^ Å), and ρ_e_ is the electron density of the material. Since ρ_e_ is on the order of 1 Å^−3^ for all materials we find that δ is in the 10^−6^ range for 1 Å wavelength. Hence, X-ray lenses are only slightly focusing due to the weak interaction of X-rays with matter. For collimation purposes focal lengths of 229 m are targeted and beryllium (Be) is preferred as lens material due to its high ablation threshold (Madsen *et al.*, 2013 ▸) and existing manufacturing technology. Investigations of different Be grades and their influence on the X-ray beam quality have been conducted and lenses manufactured accordingly (Roth *et al.*, 2014 ▸). Consequently, at the MID instrument only lenses made from hot-rolled Be ingot (IF-1 and IS50M qualities) are used. X-ray imaging using a stack of IF-1 lenses has been performed in a recent holography experiment at MID (Hagemann *et al.*, 2021 ▸) which also allows visualizing typical lens imperfections out of the focal plane. The lenses (RXOPTICS) installed in CRL-1 and CRL-2 consist of 2D parabolic reliefs, characterized by the parabola radius *R*, that are pressed into shape on both sides of a Be disk. Hence, the focal length *f* of a stack of *N* disks is (Schroer *et al.*, 2003 ▸) 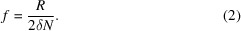
Combining several actuators of CRL-1 containing different numbers (*N*_*j*_) of lenses with different radii (*R*_*j*_), the total focal length is simply calculated as 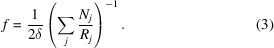
In Table 1 ▸ the configuration of CRL-1 is listed. It allows collimating the beam in a range from 5 to 25 keV (λ from 0.5 to 2.5 Å) and in addition to focus at intermediate positions along the beamline or on the sample (at ∼959 m), depending on the X-ray wavelength (Roth *et al.*, 2014 ▸).

### 2.2. Offset mirrors   

The offset mirrors M1 and M2 (at 290 and 301 m, respectively) are similar to those employed in the beam transport of SASE-1 (Tschentscher *et al.*, 2017 ▸; Sinn *et al.*, 2019 ▸). Two horizontally deflecting Si mirrors of 0.9 m active length (polished surface) are used to suppress background from higher-order harmonics. The mirrors feature three horizontal stripes from which the beam can be reflected: a Pt-coated stripe, a native Si stripe, and a B_4_C-coated stripe. After the two mirror reflections the beam must be offset 25 mm with respect to the optical axis of the undulator to bypass a heavy-duty safety absorber that prevents all other radiation, including very hard spontaneous X-rays and Bremsstrahlung, from reaching the experimental stations. Mirror settings close to the critical angle for total external reflection θ_C_ = 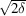 yielding the required beam offset and limiting the footprint to 0.9 m can be found for energies in the range 5–25 keV by reflecting from the right stripe. Depending on the desired footprint it can be advantageous to use the Pt stripe already from ∼14 keV and above. The second mirror M2 features a possibility for slight meridional bending to ensure a beam with symmetric cross section at the end of the tunnel.

### 2.3. High-power slits   

Slits are employed to block and absorb unwanted parts of the XFEL beam, *e.g.* parasitic scattering from upstream optics and spontaneous radiation. Furthermore, they provide an optical aperture to shape or collimate the beam. The high power of the beam leads to substantial heat load so each slit blade requires active cooling by means of a circulating water supply (capable of removing 50 W power) as well as temperature monitoring. The slit system (JJ X-Ray) consists of an UHV chamber with four motorized linear stages to translate the blades independently. Each slit blade comprises two absorbing components, an upstream 75 mm-thick B_4_C block followed by a 2 mm tungsten carbide blade. To prevent the unattenuated beam from accidentally hitting the internal mechanics, the entrance flange of the chamber is equipped with a protective B_4_C aperture of 20 mm thickness and 50 mm diameter opening. The beam transport to the MID instrument includes three such power slit units. Two of them, PSLIT1 and PSLIT2, are located in the XTD6 photon tunnel section (at 727 m and 887.5 m) and the third unit, PSLIT3, is situated in the OH at 948 m, see Fig. 1 ▸.

**Figure 2:**
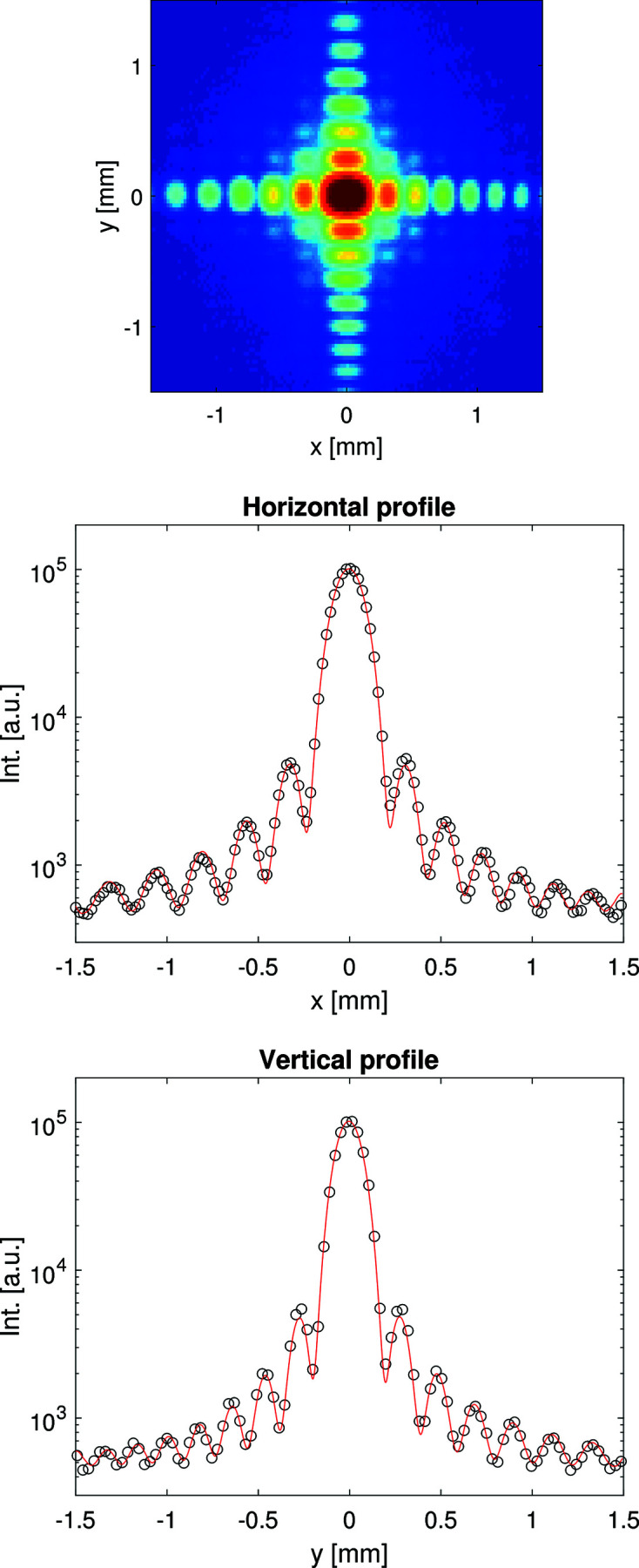
(Top) Slit diffraction pattern from PSLIT2 recorded by the imager at 936.5 m. (Middle) Horizontal cut through the data (points) and a model (red line) corresponding to a nominal opening of *a*_0_ = 31.0 µm. (Bottom) Vertical cut through the data (points) and a model (red line) corresponding to a nominal opening of *a*_0_ = 35.7 µm. Both model calculations use *z*_0_ = 99 mm; see text.

Fig. 2 ▸ shows slit diffraction from PSLIT2 recorded using a pop-in imager unit with YAG screen and camera (Koch *et al.*, 2019 ▸) located 49 m downstream. The data can be modeled using the usual formula for Fraunhofer (far-field) diffraction from an rectangular aperture with an effective opening *a* that depends on *x* (or *y*) like 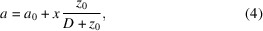
where *a*_0_ is the nominal opening, *z*_0_ is the distance between the slit blades (horizontal or vertical) along *z*, and *D* is the distance from the slit to the detector. This projection effect is always present if *z*_0_ ≠ 0 since the blades then are not in the same plane perpendicular to the beam and gives rise to the left/right and top/bottom asymmetry in the fringe pattern, see Fig. 2 ▸. *z*_0_ = 99 mm is consistent with the internal design of the slits as the distance between the tungsten carbide plate of the upstream blade and the B_4_C wedge of the downstream blade. This effect has been discussed previously for cylindrical slit blades (Le Bolloc’h *et al.*, 2002 ▸).

### 2.4. Si(111) monochromator   

In order to reduce the intrinsic bandwidth (∼10^−3^) of SASE radiation required for certain experiments, the optical layout of MID includes two X-ray monochromator devices using Si(111) and Si(220) reflections. The Si(111) monochromator is located in the photon tunnel XTD6 at 929 m and provides a bandwidth reduction to Δ*E*/*E* ≃ 1.3 × 10^−4^. The mechanical design is based on an artificial channel-cut monochromator (ACCM) concept (Shu *et al.*, 2007 ▸; Dong *et al.*, 2016 ▸). An ACCM permits the crystal surfaces to be polished individually before mounting in the crystal cage. This leads to a better surface finish than for monolithic channel-cut crystals that are more commonly used. The drawback of any channel-cut realization with channel width *h* is a variable beam offset Δ*y* which depends on the Bragg angle θ_B_ like 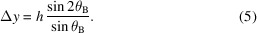
However, upstream vertical beam steering using the roll axis of M2 allows for compensating a varying Δ*y* and keeping a constant beam exit after the monochromator, regardless of the photon energy. Geometrical parameters of the Si(111) and Si(220) ACCMs, including Bragg angles and vertical offsets for two photon energies of 5 and 25 keV, are listed in Table 2 ▸. The available degrees of freedom include pitch rotations, translations, and roll motion of the crystal assembly. The rotational motion (Bragg angle for both crystals) is realized in vacuum using a linear stage acting on a sine bar assembly that houses the monochromator crystals. This arrangement limits the total stroke of the Bragg rotation to 35° but provides a very stable design. A weak link spring mechanics between the two crystals allows aligning the Bragg planes of the two crystals to be parallel, or to achieve a slight detuning thereby suppressing higher harmonics. This is all accomplished by motions of the second crystal using coarse/fine pitch and roll angular motions. The monochromator vacuum vessel is mounted on a massive support table (Huber Diffraktionstechnik) which provides vertical and horizontal translations. The optics is operated under UHV conditions and equipped with cryogenic cooling using a He cryo-compressor (Cryomech). The Si crystals are connected via braids to a cold finger and power dissipation of up to 40 W at 100 K have been observed (Dong *et al.*, 2016 ▸) which is sufficient in our case. Temperature stabilization and control is achieved by counter heaters and thermocouples installed on the crystals. First commissioning results and simulation studies of adiabatic monochromator heating during pulse trains at MID are reported elsewhere (Petrov *et al.*, 2019 ▸).

### 2.5. CRL-2 unit   

CRL-2, the second compound refractive lens unit (JJ X-Ray) is located at 931 m close to the end of XTD6 and gives the possibility of focusing the beam on the sample position reaching 1–2 µm diffraction-limited beam size (Madsen *et al.*, 2013 ▸). It is often used in combination with CRL-1 as beam collimator to achieve higher transmission. Similar to CRL-1, the device consists of ten actuator arms mounted with different Be refractive lenses as given in Table 3 ▸.

## 3. Optics hutch   

The optics hutch (OH) is located in the experimental hall at about 950 m, see Fig. 1 ▸. It can be separated from the upstream XTD6 tunnel and the downstream experimental hutch (EH) by shutters that will block the X-ray beam when closed. This means that for instance the split-and-delay line (SDL) which contains sensitive optical components and the Si(220) monochromator can remain under beam illumination conditions in OH while there is still access to the EH. This combination of stability in some areas and flexibility in others is a key element in the design of MID. The OH features a Si(220) double-crystal monochromator, a heavy-duty table carrying slit, imager and attenuator units, as well as the SDL, all described in the following.

### 3.1. Si(220) monochromator   

The second ACCM of MID, featuring a pair of Si(220) crystals, is located in OH at 946 m. The Si(220) reflection provides a bandwidth of Δ*E*/*E* ≃ 5.9 × 10^−5^. The mechanical design and cryo-cooling concept is identical to the Si(111) ACCM device with parameters listed in Table 2 ▸. The cryo-cooled Si(220) monochromator is ideal to reduce the heatload of the beam for use together with the SDL which is also based on Si(220) optics, see Section 3.4.

### 3.2. Imager   

A beam imager is located after the high-power slit unit (see Section 2.3) at 948.5 m in the OH. The device consists of a YAG:Ce scintillator screen (50 µm thickness and 40 mm × 40 mm area) mounted on a movable manipulator at 45° angle with respect to the beam direction. The image is formed by CCD camera (Basler) equipped with an objective lens and optional gray filters. Similar pop-in imagers are also located in the photon tunnels and more details are given by Koch *et al.* (2019 ▸).

### 3.3. Attenuator   

To adjust the XFEL beam intensity on the downstream optical components and the sample, the MID instrument layout includes two attenuator units (JJ X-ray). The first unit is located in XTD6 at 880 m and the second unit in OH at 949 m, see Fig. 1 ▸. The devices have identical designs consisting of a UHV chamber with four manipulator arms that can be independently inserted to intersect the beam. Each arm has seven positions: one empty slot and six different filters made of Si, B_4_C, and CVD diamond of varying thicknesses (OH attenuator configuration shown in Table 4 ▸). The filters with 20 mm × 20 mm area are mounted on a copper holder with integrated water cooling (capable of removing 20 W power) and equipped with temperature sensors. For protection purposes, a B_4_C aperture with 20 mm diameter and 25 mm thickness is mounted at the chamber entrance.

### 3.4. X-ray split-and-delay line   

**Figure 3:**
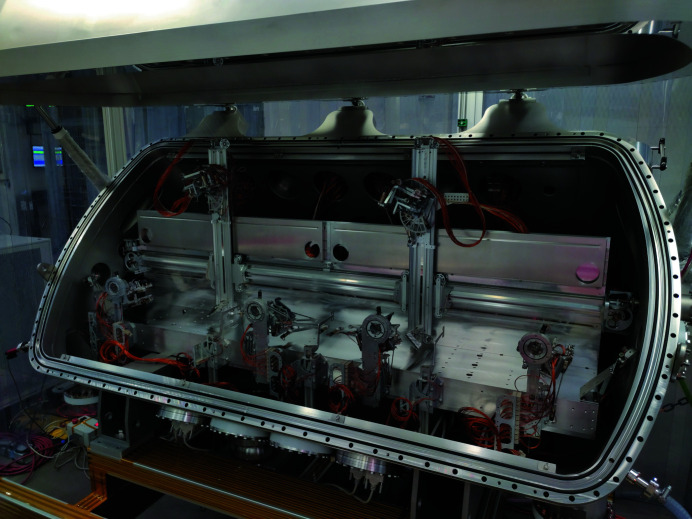
Photograph of the open split-delay line installed in MID’s OH with the mechanics and optics inside visible.

The hard X-ray split-and-delay line (SDL) for MID has been outlined in earlier publications (Lu *et al.*, 2016 ▸, 2018 ▸; Friedrich *et al.*, 2017 ▸). It is inspired by previous instruments (Roseker *et al.*, 2011 ▸; Osaka *et al.*, 2016 ▸) and recently novel ideas to improve the stability and ease of alignment of a SDL have emerged (Sun *et al.*, 2019 ▸; Li *et al.*, 2020 ▸). The SDL enables time-domain studies of ultrafast dynamics in materials by means of X-ray pump/X-ray probe measurements and X-ray speckle visibility spectroscopy (XSVS). The incoming X-ray pulse is split into two parts that are guided into different optical branches of the SDL with a path-length difference (PLD) between them that can be changed by motion of the Si(220) Bragg crystals diffracting the beam in the upper branch. Every 1 µm additional PLD results in 3.3 fs increase of Δ*t*. In XSVS two coherent split pulses scatter from the same sample volume within a short time delay Δ*t*, smaller than the integration time of the detector. Using a contrast analysis of the summed speckle intensity pattern *I*_res_ = *A*_1_ *I*(*t*) + *A*_2_ *I*(*t* + Δ*t*) it is possible to infer whether the sample structure changed during Δ*t* or remained static (Gutt *et al.*, 2009 ▸). Here *r* = *A*_1_/*A*_2_ is the intensity ratio between the split pulses produced by the SDL (ideally *r* = 1). By scanning the split pulse delay Δ*t* the second-order intensity autocorrelation function *g* ^(2)^ can be mapped out following 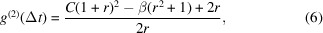
where the measurable quantities are 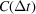 = 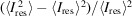, the normalized variance (speckle contrast) of the summed speckle pattern, and β = (〈*I* ^2^〉 − 〈*I*〉^2^)/〈*I*〉^2^ the speckle contrast of a single image. *g* ^(2)^ is also accessible by regular X-ray photon correlation spectroscopy (XPCS) (Grübel *et al.*, 2007 ▸; Madsen *et al.*, 2020 ▸) in sequential mode with 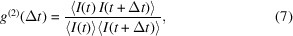
but here the smallest accessible Δ*t* is limited by the detector speed thus preventing ultrafast studies. For XSVS a co-linear propagation of the two split pulses after the SDL is advantageous to aim at the same scattering volume of the sample. A SDL can also be used for pump–probe experiments where an inclination (non co-linear mode) between the two pulses is preferred in some cases (van Thor & Madsen, 2015 ▸). This mode is also provided by the device at MID in combination with the experiment hutch mirror. The SDL has been installed in winter 2019/2020, see Fig. 3 ▸, and is expected to be available for experiments in 2021.

## 4. Experimental hutch   

The experimental hutch (EH) of MID is about 9 m wide and 17 m long with a shape guided by the high-quality floor (PILLONI GIOVANNI) on which motion of the long 2θ arm and detector table, both with weights of several tons, is possible using stiff air-pads. The high-quality floor has a planarity better than 600 µm over the entire ∼66 m^2^ surface and a roughness (r.m.s.) smaller than 10 µm over the typical footprint of an air-pad (size 400 mm). The ceiling height of EH is 4 m which allows installation of tall equipment like cryostats that can be manipulated with the hutch cranes.

### 4.1. Experimental hutch mirror system   

**Figure 4:**
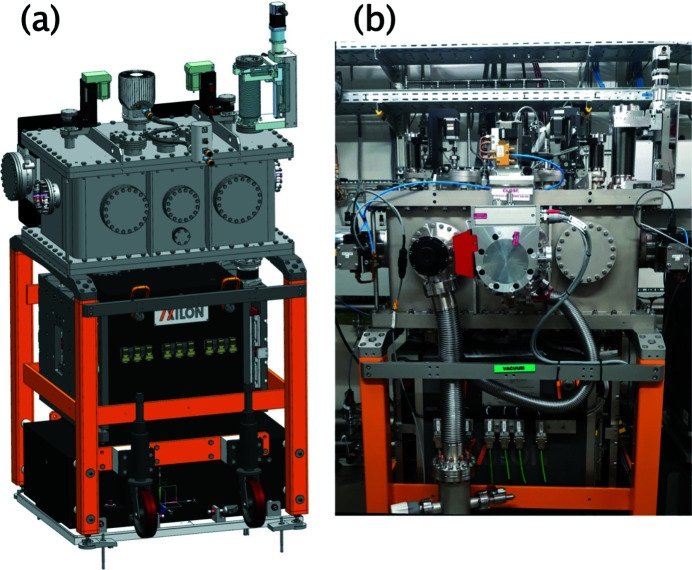
Dual X-ray mirror: (*a*) design drawing; (*b*) photograph of the device installed in the experimental hutch of MID.

A dual X-ray mirror system referred to as the Experimental Hutch Mirror (EHM), see Fig. 4 ▸, is installed in the EH at 955 m (Fig. 1 ▸) which is about 4 m upstream the sample position. The EHM (AXILON) is designed for two types of experiments, the first one being X-ray pump/X-ray probe investigations enabled by the SDL (see Section 3.4). The SDL can produce two vertically offset X-ray beams where the lower beam is delayed by up to 800 ps with respect to the upper one. In order to have both beams overlapping at the sample the lower beam must be deflected upwards. This is possible by using the EHM which features an upwards reflecting X-ray mirror. The second use case is X-ray scattering experiments on liquid surfaces where the X-ray beam is applied under grazing-incidence conditions. This is achieved by a downwards reflection mirror inside the EHM. Hence, the EHM features two identical X-ray mirrors placed one above the other and with the two reflecting surfaces facing each other. A vertical offset (gap) between them enable unhindered beam passage when the device is not in use. Each mirror consists of a flat profile Si substrate of 500 mm length and 40 mm width (Zeiss) which is coated with B_4_C and Pt stripes (Störmer *et al.*, 2018 ▸; Vannoni *et al.*, 2019 ▸). The mirrors are mounted on a high-precision motorized mechanical support inside the UHV chamber. To minimize vibrations, the mirror mechanics is resting on a three-point kinematical mount supported by a massive granite equipped with a vertical jack adjustment unit (40 mm stroke) that carries the in-vacuum mechanics and is decoupled from the vacuum vessel. The in-vacuum mechanics is designed to have two individual cradles that provide independent precise alignment of pitch and roll tilts of each mirror. Horizontal translation of the in-vacuum mechanics (30 mm stroke) enables the choice of mirror stripe depending on experimental demands. To manage the high-heat-load conditions at MHz operation of EuXFEL, the EHM design maintains the Si mirrors at cryogenic temperatures and includes thus heat shielding, cooling interfaces, copper antlers and braids, as well as integrated Pt100 sensors and heaters (Waterstradt, 2015 ▸) which provide stable operation conditions using a pulsed-type He cryo-compressor (Cryomech), similar to the monochromator design. For alignment purposes built-in diagnostics are mounted to the top flanges of the vacuum vessel. This includes upstream and downstream horizontal slits (100 µm gap) and a Si photodiode (10 mm × 10 mm active area) that can be translated in/out of the beam.

### 4.2. Differential pumping section   

The differential pumping section DPS (Cinel) separates the upstream UHV section of the MID instrument from the HV section downstream. It consists of three individual chambers each equipped with a turbo pump (Pfeiffer). The X-ray beam passes from one chamber to the other via 4 mm × 40 mm (h × v) slits manufactured in B_4_C blocks. In addition, two X-shaped B_4_C masks can narrow down the opening even further but still allow the passage of two split beams from the SDL. The masks are motorized in *x* and *y* and can be adjusted depending on the beam geometry. The DPS protects sensitive optical components, like the monochromator and SDL in the OH as well as the mirror chamber in the EH, from pollutants potentially originating from the sample chamber. The DPS can operate with pressures on the HV side of up to 0.1 mbar while still keeping 10^−8^ mbar, or better, on the UHV side. Pre­alignment of the entire DPS vessel is possible in *x* and *y* as well as for the yaw.

### 4.3. Multi-purpose chamber   

The multi-purpose chamber MPC (PINK Vakuumtechnik) consists of a steel vacuum vessel mounted on a heavy granite base bolted to the floor. Internal mechanics of the MPC allows control of optics in order to focus the X-ray beam spot to below 1 µm diameter as well as high-precision positioning and scanning of the sample to interact with the X-ray and optical laser beams (optional). All interior components are directly connected to the granite via mechanical feedthroughs and thereby decoupled from the vessel itself to prevent environmental vibrations propagating to the sensitive parts. Depending on the experimental configuration, the sample chamber can be either equipped with a DN500 exit flange or, in wide-angle X-ray scattering (WAXS) configuration, a curved Kapton window as well as a flange connection for the transfer tube leading to the diagnostic end-station. In small-angle X-ray scattering (SAXS) geometry, the MPC is directly connected to the downstream flight path and detector hence providing a windowless configuration. The operation of two turbo pumps (Pfeiffer) ensures a constant vacuum level of the MPC even when outgassing or evaporating sample systems (*e.g.* liquid jets) are used.

Providing the option of final beam shaping close to the sample position, the MPC interior hosts a small hexapod (H-811, Physik Instrumente) for nano-focusing CRLs (Seiboth *et al.*, 2017 ▸) or other optics with short focal length. The hexapod is mounted on an optical bench which allows up to 430 mm translation along the beam and ±20 mm in the perpendicular directions, both motions having 1 µm resolution. This is important for adjusting the focal point precisely with respect to the sample position. Focused beam sizes less than 300 nm × 300 nm have been achieved. To prevent any temperature-induced instabilities all motors are actively cooled which is especially important under vacuum conditions.

The sample stage inside the MPC consists of a larger hexapod (H-840, Physik Instrumente) mounted on a height translation (±40 mm travel range, 1 µm resolution) and a ±100° rotation stage with 5 µrad resolution, or better (Huber Diffraktionstechnik). The maximum load is 40 kg and the top plate of the hexapod is water cooled. The nominal beam height over the top plate is 170 mm which leaves enough space for hosting different kinds of sample environment. Most motions inside the MPC are equipped with high-resolution encoders (linear or rotation).

The MPC vessel offers flanges of different size for in-vacuum implementation of ancillary sample environment that is not fitting on the sample hexapod, *e.g.* a furnace, a cryostat, or liquid jets. Particularly useful is the vertical DN160 flange on top of the chamber directly above the sample hexapod, as well as a horizontal DN200 flanges on the side pointing perpendicular to the beam and centered at the nominal beam height.

An in-air option is provided if the experiment or sample are not compatible with vacuum or if the sample environment cannot fit inside nor be attached to the MPC feed-through flanges. In this case, the top part of the MPC vessel (lid) is detached and removed providing access to the optical bench and hexapods inside that are fully operational in air. To separate the open MPC from the vacuum beamline upstream, a diamond window can be installed between DPS and MPC. This diamond window features a 3 mm × 3 mm defect-free HPHT type IIa diamond crystal of 200 µm thickness brazed into a larger CVD wafer (Diamond Materials). The window is water cooled and since the beam passes through the defect-free area no wavefront distortions are observed. Downstream of the sample a second diamond window is used as entrance to the flight path leading to the detector (SAXS configuration) or to the transfer beam pipe connected to the diagnostic end-station about 10 m downsteam. The area detector of choice for MHz data acquisition is the Adaptive Gain Integrating Pixel Detector (AGIPD), see Section 4.5. The large-field-of-view configuration where the detector is very close to the sample is not compatible with in-air MPC operation due to the lack of a large vacuum window for the detector.

### 4.4. Experimental configurations   

XSIS, the X-ray Scattering and Imaging setup (PINK Vakuumtechnik), is the combination of MPC, detector arm, and detector support and was designed in collaboration with ESRF (Grenoble, France). The aim is to enable a multitude of experimental configurations for the different use cases of MID.

**Figure 5:**
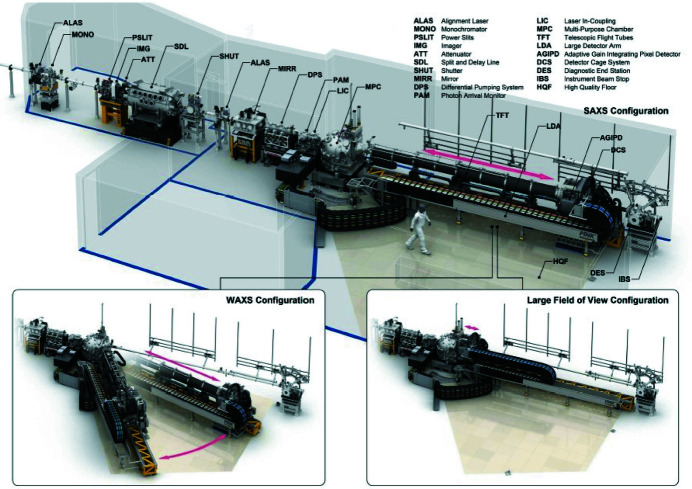
3D CAD-models of MID’s optics and experiment hutches and the components in SAXS geometry. The insets at the bottom show the instrument in two other configurations – WAXS and LFOV. Credit: European XFEL/Rey.Hori

The XSIS can be operated in three different configurations (see Fig. 5 ▸) depending on the requirement to the detector field-of-view, the spatial resolution, and the angular range:

(i) Large field-of-view configuration (LFOV).

(ii) Small-angle X-ray scattering (SAXS) configuration.

(iii) Wide-angle X-ray scattering (WAXS) configuration.

**Figure 6:**
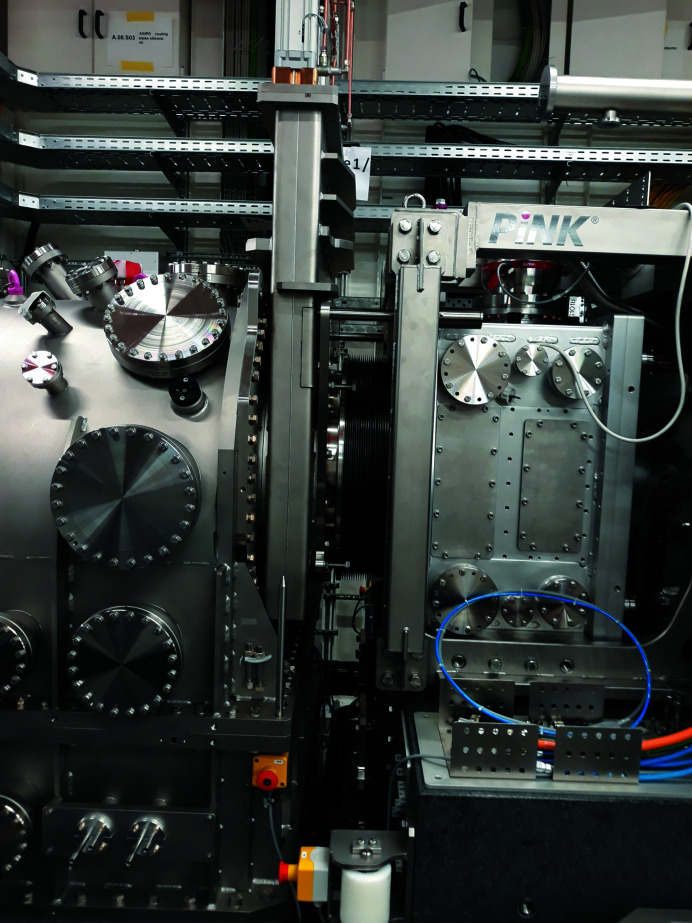
Photograph of the MID instrument in LFOV configuration with AGIPD attached to the MPC.

In LFOV configuration AGIPD is directly connected to the MPC. They share the same vacuum and the detector can be moved very close to the sample reaching distances down to ∼20 cm (see Fig. 6 ▸). In this case, the AGIPD vacuum vessel is attached, via a 500 mm-diameter gate valve (DN500), to the large exit flange of the MPC. With an open valve the detector head can be translated into the MPC and brought close to the sample hexapod. In the retracted position, the gate valve can be closed and the detector sensors are protected. The central hole of AGIPD allows unhindered transmission of the direct beam to the diagnostic end-station (DES, see Section 4.6) via a vacuum beam pipe connected to the back side of the AGIPD vessel. Scattering angles up to 2θ ≃ 23° can be reached in LFOV. Examples of scattering data taken in LFOV can be seen later in Figs. 10(*e*) and 10(*f*).

**Figure 7:**
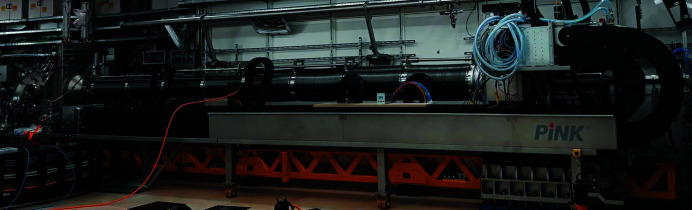
Photograph of the the SAXS configuration with AGIPD located at the maximum distance of ∼8 m from the sample.

In SAXS, similar to the LFOV configuration, the detector arm is positioned in the forward direction but with a flexible bellow system between MPC and AGIPD as shown in Figs. 7 ▸ and 8 ▸. Here, sample-to-detector distances between 3 m and 8 m are accessible and can be readily varied during an experiment without venting the system. The SAXS configuration is windowless, similar to LFOV. Examples of scattering data taken in SAXS geometry are shown in Figs 10(*a*)–10(*d*).

The flexibility in changing sample–detector distance is provided by use of eight vacuum bellow sections with inner diameters between 300 and 400 mm. The motion is realized by three servomotors driving a rack-and-pinion system on the long detector arm – two motors driving the detector table and one motor supporting the motion of the bellow system. Additional clamping brakes support the holding force of the motors and ensure stability when the motion is stopped. A second pipe and bellow system (DN100) connects the back side of AGIPD to the diagnostic end-station at the end of the EH, see Section 4.6.

**Figure 9:**
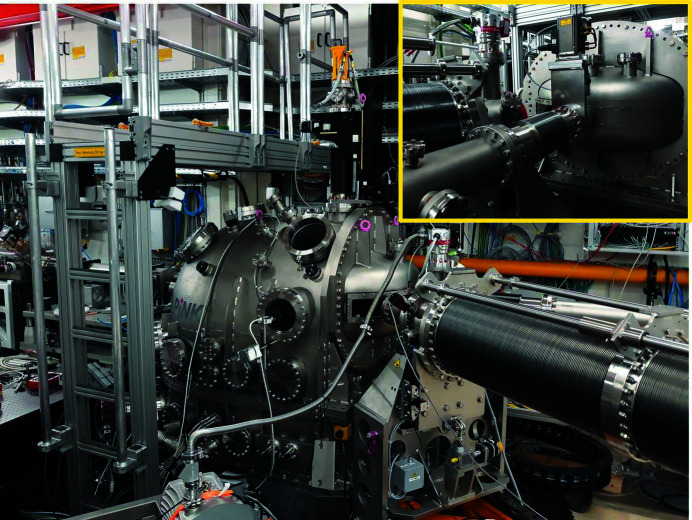
Photograph of the MID instrument in the WAXS configuration. The large DN500 exit flange used in LFOV and SAXS is replaced by a rectangular Kapton window allowing the scattered beam to pass. Inset: close-up on the transfer tube letting the direct beam through to the DES.

In WAXS geometry the detector table and arm can be rotated around the sample position on the high-quality floor covering scattering angles of 2θ = 14°–55° (see Fig. 9 ▸). In contrast to the two other configurations, WAXS is not windowless as two 500 µm-thick Kapton windows are required in the path of the scattered beam for vacuum separation. Similar to the SAXS configuration, the sample-to-detector distance is continuously adjustable between 3 and 8 m. After interaction with the sample, the direct transmitted beam (if any) propagates through a windowless vacuum pipe and bellow system connecting MPC and DES. The 2θ rotation of the long detector arm is realized by a motorized rubber wheel connected to the granite supporting the detector cage. When the detector is moved radially or the arm and detector are rotated, they hover over the high-quality floor by an air gap of ∼20 µm provided by high-pressure stiff airpads.

### 4.5. AGIPD   

The AGIPD (Allahgholi *et al.*, 2015 ▸, 2019 ▸) is the primary detector of the MID instrument. It consists of 16 modules, each with 512 × 128 pixels, grouped into four independently movable quadrants. The pixel size is 200 µm × 200 µm in a 500 µm-thick silicon sensor material. A central hole allows the direct beam to pass through the detector towards the DES. Two distinctive features make the AGIPD detector especially suitable for experiments at European XFEL. First, each pixel has its own storage cells (analog, grouped in 11 rows of 32 cells each) which can be filled with up to 352 acquisitions at rates up to 4.5 MHz. This makes the detector capable of resolving single bunch intensity images in the high repetition mode of EuXFEL. The subsequent read-out of all storage cells happens at 10 Hz rate between pulse trains. Additionally, each pixel can automatically switch between three gain stages, therefore adapting the gain to the scattering signal level. With this, a high dynamic range from single photon sensitivity, up to 10^4^ photons at 12.4 keV, can be achieved. An automatic data calibration pipeline is available to the users, which corrects the raw AGIPD data for known offsets and gains. Additionally, more sophisticated corrections were implemented during the course of operation, correcting for signal-dependent baseline shifts and storage cell dependent gains in high-gain mode. Furthermore, a common mode correction was implemented, which identifies empty pixels and corrects for slow, long-term drifts of single pixel offsets within blocks of 32 storage cells and pulse-to-pulse fluctuations of the baseline within one ASIC.

**Figure 10:**
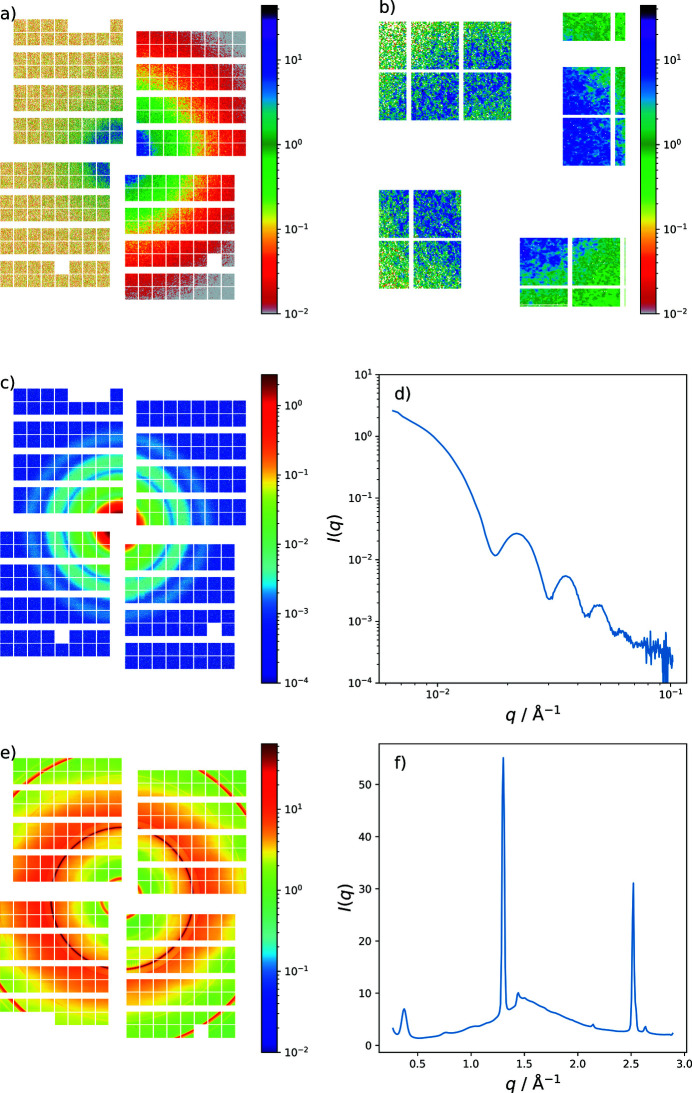
(*a*) Coherent small-angle scattering pattern (speckle) from Vycor measured by the AGIPD. The left two quadrants display scattering by a single SASE pulse (∼50 fs duration) while the right-hand side shows the average over 25000 acquisitions. (*b*) Magnification of the scattering ring of Vycor shown in (*a*) at low *q*-values. (*c*) Small-angle scattering of silica spheres (25 nm radius) dispersed in water illustrating the *q*-range covered (9 keV and 7.8 m). (*d*) Radial integration of the scattering image shown in (*c*). (*e*) Powder diffraction of LTO taken in LFOV configuration (9.3 keV and 24 cm) and radial integration shown in (*f*). AGIPD consists of 256 (16 × 16) square units (ASICs) of 12.8 mm × 12.8 mm size (64  ×  64 pixels).

Exemplary scattering data sets recorded by AGIPD in SAXS and LFOV are displayed in Fig. 10 ▸. A coherent scattering pattern (speckle) from a porous silica scattering standard (Vycor) measured with 9 keV photons at a sample–detector distance of 7.8 m is shown in Fig. 10 ▸(*a*). The speckle pattern can be observed over the full area of the detector, demonstrating the high sensitivity. All color scales show photons per pixel. Fig. 10 ▸(*b*) is a magnification of the scattering ring of Vycor at low *q*-values where the speckle pattern is readily seen. The left halves of Figs. 10(*a*) and 10(*b*) ▸ show single pulse data while the right sides are averaged over 25000 pulses. Figs. 10(*c*) and 10(*d*) ▸ show the small-angle scattering of silica spheres (25 nm radius) dispersed in water to illustrate the typical *q*-range covered. Smaller *q*-values might become accessible by further decreasing the gap between detector modules. In Figs. 10(*e*) and 10(*f*) ▸ the powder diffraction data of lithium titanate (LTO) taken in LFOV configuration are shown.

### 4.6. Diagnostic end-station   

**Figure 11:**
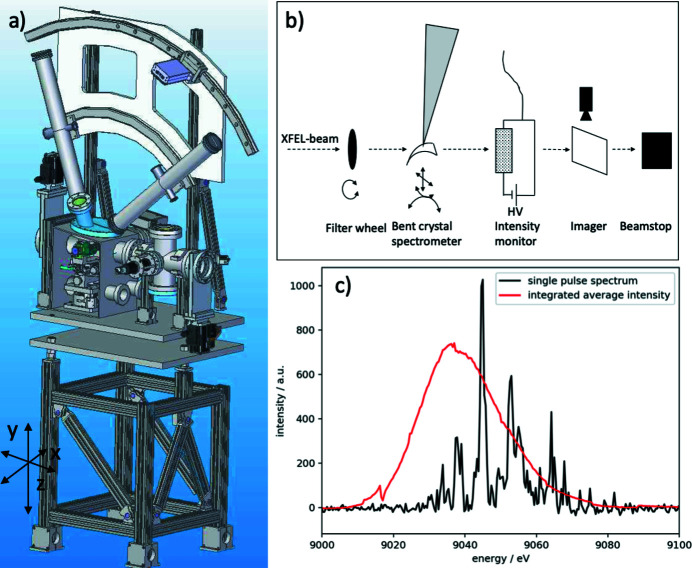
(*a*) 3D CAD-model of the DES. (*b*) Conceptual sketch of the elements inside DES. (*c*) Single pulse and average spectra measured with the bent diamond spectrometer.

A diagnostic end-station (DES) equipped with a spectrometer, intensity detectors, an imaging unit, and a beam stop is positioned at the end of the MID instrument at about 969 m, see Fig. 1 ▸. It provides tools for characterization and overall alignment of the beam and samples. For samples placed in the MPC with a sufficiently high transmission the DES can be used in parasitic mode providing online diagnostics. DES consists of a compact vacuum chamber with all devices integrated. A drawing and a functional sketch of DES are displayed in Figs. 11(*a*) and 11(*b*) ▸, respectively. The main feature of DES is a bent diamond crystal spectrometer operating in dispersive vertical scattering geometry (Zhu *et al.*, 2012 ▸; Boesenberg *et al.*, 2017 ▸, 2019 ▸; Samoylova *et al.*, 2019 ▸; Kujala *et al.*, 2020 ▸). For full flexibility, up to four different crystals can be mounted simultaneously and for two of these a flexible motorized bending is foreseen. The Bragg (θ) rotation stage for the spectrometer crystals has a precision better than 0.1 mdeg to align reflection geometries with a narrow Darwin width. The diffracted signal is recorded by a Gotthard-I strip detector of 50 µm pitch (Mozzanica *et al.*, 2012 ▸) with single pulse resolution enabled by charge storage cells (currently limited to 120 pulses train^−1^ at 500 kHz repetition rate). The X-rays diffracted by the crystal are transmitted through a Kapton window and absorption losses are minimized by an evacuated flight tube leading to the Gotthard detector located 1 m from the bent crystal. A future upgrade will include a 25 µm pixel-pitch Gotthard-II detector (Zhang *et al.*, 2018 ▸) matching the maximum repetition rate (4.5 MHz) of the EuXFEL. A 20 µm thin bent diamond spectrometer with a radius of curvature of ∼90 mm is mounted in (220) reflection geometry. Typical spectra of a single pulse (black line) and an average (red line) of the SASE radiation can be found in Fig. 11(*c*) ▸. Monitoring the spectra of SASE pulses is not only important for spectroscopy applications but can also be used to estimate the X-ray pulse duration from the energy width of the SASE spikes (Inubushi *et al.*, 2012 ▸). To perform an energy calibration of the spectrometer a filter wheel at the DES entrance is equipped with foils of different materials that can be inserted into the beam. The imaging unit is based on 50 µm thin YAG crystals that convert the X-rays into visible light. An optical camera (Basler) operating at 10 Hz repetition rate records the YAG signal. A high- and low-resolution option (about 3 and 9 µm) is realized by two different objectives mounted on the cameras. To avoid image distortions in high resolution, the YAG screen is mounted perpendicular to the beam and combined with an optical mirror in 45° geometry. The imagers are important diagnostic tools for verification of beam pointing but they also facilitate alignment of upstream X-ray optical components, nano-focusing lenses, and the sample. In addition, the DES is used as a testbed for characterization of diamond-based intensity detectors operating like solid state ionization chambers (Roth *et al.*, 2018 ▸). Single pulse intensity measurements up to 4.5 MHz repetition rate of the European XFEL are possible with these detectors. The instrument beam stop at the end of DES consists of a sandwich of 40 mm B_4_C, 10 mm Al, and 15 mm steel and marks the end of MID at ∼970 m.

## 5. Commissioning results   

### 5.1. X-ray photon correlation spectroscopy   

We demonstrate the MHz XPCS capabilities (Lehmkühler *et al.*, 2020 ▸) of the MID instrument on a colloidal sample system of silica nanoparticles (*R* = 10 nm) dispersed in water. SAXS acquisitions were obtained with the AGIPD detector in SAXS geometry at a sample–detector distance of 7.8 m and a photon energy of 9 keV. The repetition rate of the EuXFEL and the AGIPD detector were 2.2 MHz and 60 scattering patterns were recorded per train. The data were corrected using the data correction pipeline described earlier.

Special care was taken to exclude radiation damage effects on the measurement. In order to achieve a good compromise between speckle contrast and radiation dose on the sample, the beam size was set to 10 µm by using the nanofocusing CRL stack located in the MPC sample chamber about 30 cm upstream of the sample in an out-of-focus condition. The beam intensity was adjusted using solid state attenuators located in the XTD1 tunnel and the MID optics hutch (Section 3.3). A quartz capillary containing the sample was constantly moved during the acquisitions with a speed of 400 µm s^−1^, resulting in 40 µm separation between two trains impinging the sample. The movement of the sample between two pulses (444 ns apart) is 18 nm which is small compared with the beam size and can be neglected.

**Figure 12:**
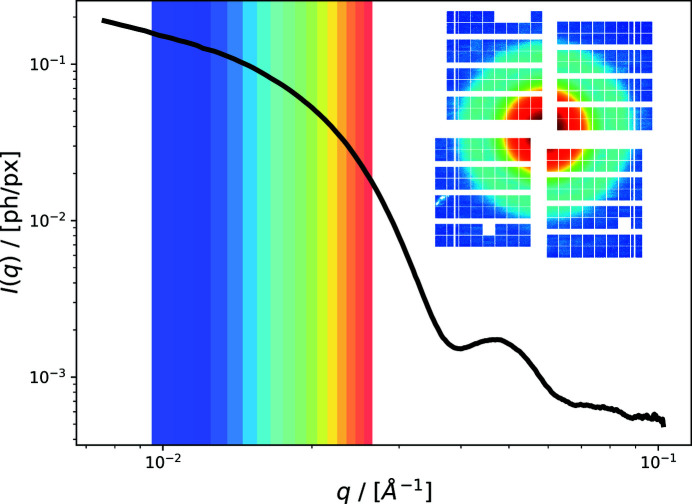
Averaged small-angle X-ray scattering data of an aqueous silica nanoparticle solution. Color-shaded regions indicate the *q*-bins for which autocorrelation functions are calculated. The inset shows the mean scattering intensity for each pixel of AGIPD.

Figure 12 ▸ displays the azimuthally integrated scattering intensity of the nanoparticle solution, averaged over 1800 trains with 60 pulses each. The shaded regions mark the *q*-bins for which an autocorrelation function was calculated. The inset shows the mean intensity per pixel on the AGIPD detector.

**Figure 13:**
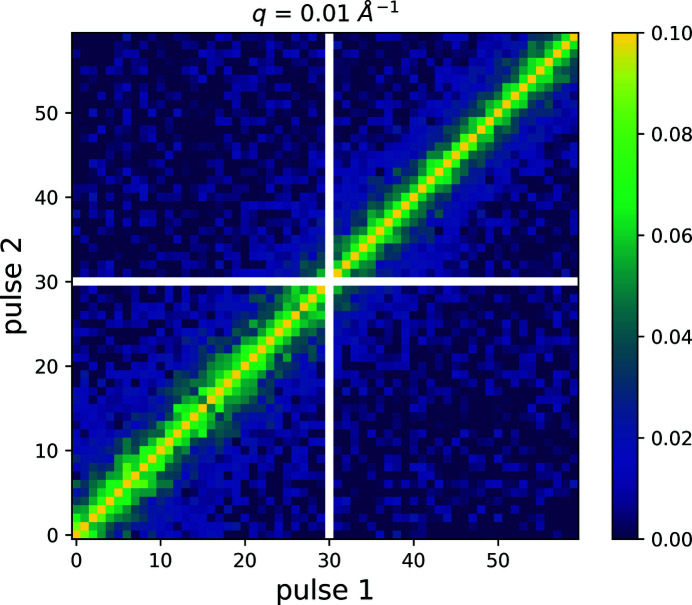
Two-time correlation function calculated for *q* = 0.01 Å^−1^ and averaged over 1800 pulse trains. Pulse number 30 was saved in a storage cell of AGIPD which, due to non-ideal behavior, was excluded from further analysis and hence masked in this representation (white cross).

Pixels with non-uniform response are identified and subsequently masked by first calculating the mean intensity of each pixel and storage cell and afterwards calculating from this a standard deviation of the mean over the storage cells for each pixel. Outliers are identified by comparing with the median standard deviation of all pixels grouped in one *q*-bin. Each *q*-bin contains 1000–7000 pixels and the two-time correlation function is calculated as 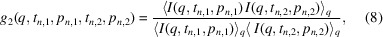
with 〈…〉_*q*_ denoting the average over all pixels belonging to one *q*-bin. *t*_*n*_ and *p*_*n*_ denote the train and pulse number, respectively. The MHz two-time correlation function is consequently calculated by correlating pulses within one train and averaging over all trains as 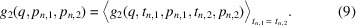
This is plotted in Fig. 13 ▸ for *q* = 0.01 Å^−1^. Due to slightly different responses of the various storage cells of a single pixel, off-train correlations were calculated from the same dataset as 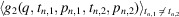 and subtracted as baseline to get rid of non-uniform detector response. This correction is possible due to the continuous movement of the sample which implies that data from successive trains are uncorrelated.

**Figure 14:**
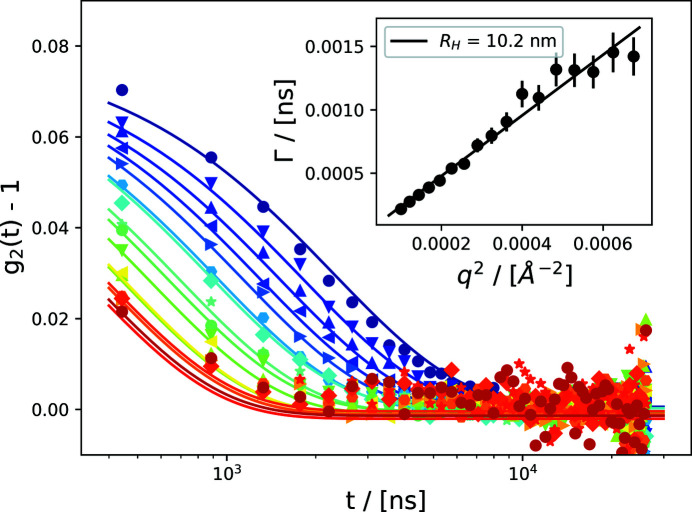
Autocorrelation functions *g*_2_(*q*,*t*) of 10 nm silica nanoparticles dispersed in water. The inset shows the dependence of the decay rate Γ as a function of *q* ^2^, illustrating diffusive behavior of the colloidal dispersion. The colorscale reflects *q* as given in Fig. 12 ▸.

It is evident from the two-time correlation function that equilibrium dynamics are probed and that no change of dynamics over the pulse train is observed. The resulting autocorrelation functions *g*_2_(*q*,*t*) are displayed in Fig. 14 ▸, where *t* denotes the time-delay between two pulses. The data are fitted assuming diffusive Brownian motion, resulting in a single exponential form,
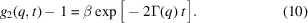
The inset shows the resulting dispersion relation, which can be well described by a linear dependence of Γ on *q* ^2^, resulting in a hydrodynamic radius of *R*_H_ = 10.2 nm, as expected.

## 6. Conclusions and outlook   

We have presented the design and capabilities of the MID instrument at EuXFEL which specializes in scattering and imaging experiments using coherent, hard X-ray FEL pulses. The high repetition rate and pulse-train structure provide novel opportunities for studies of dynamics, *e.g.* through MHz XPCS. Examples of commissioning results are shown and the instrument is open for proposals. Recently, the first results of a user experiment have been published (Hagemann *et al.*, 2021 ▸). Further commissioning results focusing on the SDL and pump–probe experiments using the femtosecond optical laser system will be communicated later.

## Figures and Tables

**Figure 8:**
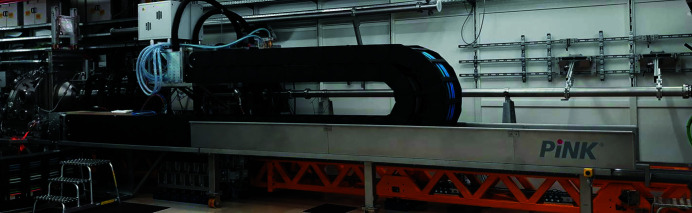
Photograph of the SAXS configuration where the bellows between MPC and AGIPD are fully compressed reducing the sample-to-detector distance to ∼3 m.

**Table:** 

Actuator	1	2	3	4	5
Lenses (*N*, *R*) (mm)	Empty	(1, 5.8)	(1, 4.9)	(1, 4.0)	(1, 3.3)
*N*/*R* (mm^−1^)	–	0.172	0.204	0.25	0.303

**Table:** 

Actuator	6	7	8	9	10
Lenses (*N*, *R*) (mm)	(2, 5.8)	(3, 4.0)	(7, 4.0)	(7, 2.0)	Empty
*N*/*R* (mm^−1^)	0.345	0.75	1.75	3.5	–

**Table 2:** Parameters of ACCM monochromators

		*E* = 5 keV		*E* = 25 keV	
ACCM type	*h* (mm)	θ_B_ (°)	Δ*y* (mm)	θ_B_ (°)	Δ*y* (mm)
Si(111)	5.5	23.3	10.1	4.5	11.0
Si(220)	7.8	40.2	11.9	7.4	15.5

**Table:** 

Actuator	1	2	3	4	5
Lenses (*N*, *R*) (mm)	None	(1, 5.8)	(2, 5.8)	(4, 5.8)	(7, 5.8)
*N*/*R* (mm^−1^)	None	0.172	0.345	0.689	1.21

**Table:** 

Actuator	6	7	8	9	10
Lenses (*N*, *R*) (mm)	(10, 4.0)	(10, 2.0)	(10, 1.0)	(10, 0.5)	None
*N*/*R* (mm^−1^)	2.5	5	10	20	None

**Table 4:** Absorber filters in the OH attenuator *d* is the filter thickness in mm.

Arm 1 (upstream)			Arm 2			Arm 3			Arm 4 (downstream)		
Slot	Type	*d*	Slot	Type	*d*	Slot	Type	*d*	Slot	Type	*d*
1	Empty		1	Empty		1	Empty		1	Empty	
2	CVD	0.8	2	CVD	0.4	2	CVD	0.2	2	CVD	0.1
3	CVD	3.2	3	CVD	1.6	3	CVD	0.8	3	CVD	0.4
4	Si	6.4	4	Si	3.2	4	Si	1.6	4	Si	0.2
5	Si	1.6	5	Si	0.4	5	Si	0.4	5	Ge	0.5
6	Si	0.8	6	Si	0.8	6	Si	0.2	6	Si	0.8
7	Si	0.2	7	Si	0.1	7	Si	0.05	7	Si	0.025
